# Phosphorescent Ir(III) complexes conjugated with oligoarginine peptides serve as optical probes for in vivo microvascular imaging

**DOI:** 10.1038/s41598-021-84115-x

**Published:** 2021-02-26

**Authors:** Mami Yasukagawa, Aya Shimada, Shuichi Shiozaki, Seiji Tobita, Toshitada Yoshihara

**Affiliations:** Department of Chemistry and Chemical Biology, Graduate School of Science and Technology, Gunma University, 1-5-1 Tenjin-cho, Kiryu, Gunma 376-8515 Japan

**Keywords:** Imaging, Microscopy, Optical spectroscopy, Photochemistry

## Abstract

Imaging the vascular structures of organ and tumor tissues is extremely important for assessing various pathological conditions. Herein we present the new vascular imaging probe BTQ-R_n_ (n = 8, 12, 16), a phosphorescent Ir(III) complex containing an oligoarginine peptide as a ligand. This microvasculature staining probe can be chemically synthesized, unlike the commonly used tomato lectins labeled with a fluorophore such as fluorescein isothiocyanate (FITC). Intravenous administration of BTQ-R_12_ to mice and subsequent confocal luminescence microscope measurements enabled in vivo vascular imaging of tumors and various organs, including kidney, liver and pancreas. Dual color imaging of hepatic tissues of living mice fed a high-fat diet using BTQ-R_12_ and the lipid droplet-specific probe PC6S revealed small and large lipid droplets in the hepatocytes, causing distortion of the sinusoidal structure. BTQ-R_12_ selectively stains vascular endothelium and thus allows longer-term vascular network imaging compared to fluorescent dextran with a molecular weight of 70 kDa that circulate in the bloodstream. Furthermore, time-gated measurements using this phosphorescent vascular probe enabled imaging of blood vessel structures without interference from autofluorescence.

## Introduction

Blood vessels form a network throughout the body, carrying blood which delivers oxygen and nutrients to tissues and removing CO_2_ and waste products. Disruption of the vascular system is closely associated with various diseases. For example, in cancer and diabetic retinopathy, abnormal angiogenesis results in an immature and heterogeneous vascular network^1^. Therefore, the visualization of vascular networks in tissues is highly important for understanding vascular diseases. Many imaging techniques, such as magnetic resonance imaging (MRI)^2^, computed tomography (CT)^3,4^, positron emission tomography (PET)^5^, photoacoustic imaging^6^ and optical imaging^7^ have been used to visualize the network of blood vessels in tissues and bodies. Fluorescence imaging based on confocal laser scanning microscopy and two-photon microscopy^8,9^ offers several advantages over these other techniques, including high spatial resolution, good sensitivity, and the possibility of multicolor imaging of blood vessels and tissue cells.

To visualize the network of blood vessels based on fluorescence imaging, many fluorescent vasculature probes have been developed using biocompatible polymers, proteins and nanoparticles^10–12^. Fluorescent dextrans, in which fluorescent dyes such as coumarin^13^, fluorescein^14^ or rhodamine derivatives^15^ are covalently bonded to dextran with a molecular weight of 70 kDa or more, are widely used as blood vessel imaging reagents. These probes have a large molecular weight, similar to that of serum albumin, and thus circulate in the bloodstream for a long time without being filtered by the glomeruli in the kidneys. Another approach is to conjugate a fluorophore to *Lycopersicon esculentum* (tomato) lectin^16,17^, which has selective affinity to *N*-acetyl-d-glucosamine, and to a fluorescent antibody^18^ with affinity to CD31, a typical endothelial marker. This approach allows the visualization of blood vessels by the binding of tagged lectin and antibody to vascular endothelial cells. Although these fluorescent probes allow visualization of the vascular network in tissues, it is often difficult to clearly distinguish between the autofluorescence of tissue cells and probe signals. Furthermore, fluorescent dextrans have much larger molecular weights than small molecule probes and thus a larger amount (by mass) must be administered to visualize the vascular network in tissues.

We have been developing optical oxygen probes based on Ir(III) complexes for in vivo oxygen measurements^19^. The oxygen status of tissue cells is maintained by the balance of oxygen supply by the blood and its consumption in cells. Thus, imaging the vascular network is important for understanding oxygen distribution throughout tissues. We recently developed hydrophilic Ir(III) complexes bearing a biocompatible hydrophilic unit, polyethylene glycol, and achieved intravascular O_2_ imaging in living tissues^20^. However, these complexes are transferred to the urinary space within 1 h of administration to mice by filtration by the glomeruli in the kidneys. Vascular imaging probes that stain vessel walls are required for long observation periods. During our previous work, on the development of a ratiometric oxygen probe^21^, we found that probes incorporating oligoarginine peptides administered intravenously to mice selectively stain the vascular endothelium, likely due to binding of the oligoarginine peptide to proteoglycans on the surface of vascular endothelial cells^22,23^.

**Figure 1:**
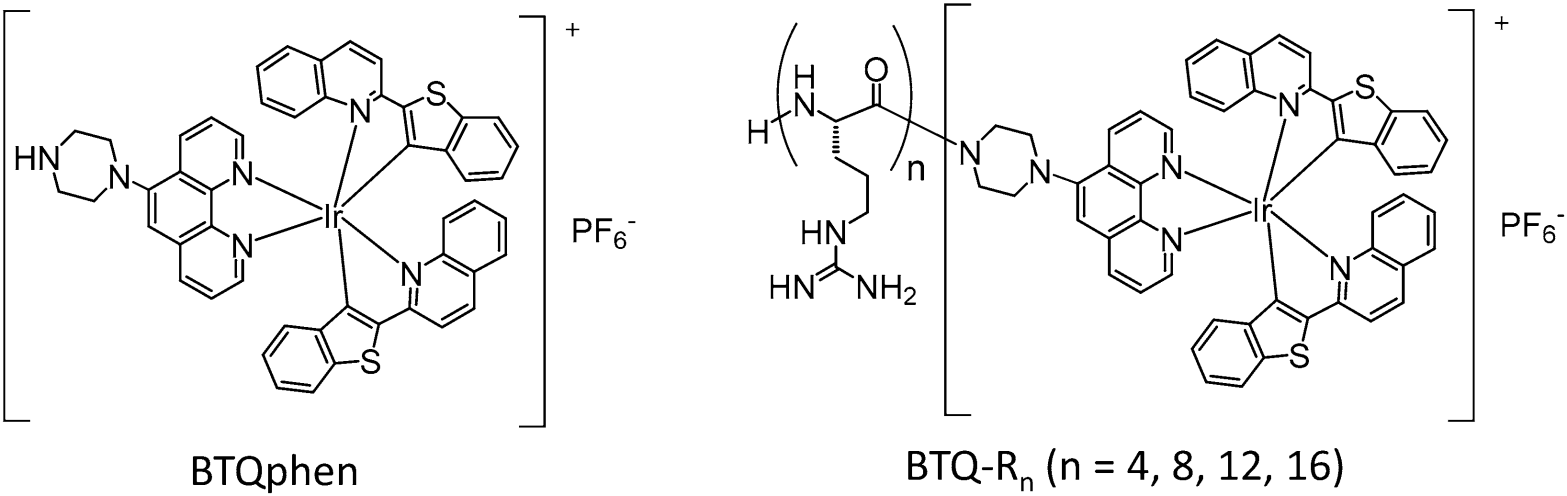
Chemical structures of BTQphen and BTQ-R_n_ (n = 4, 8, 12, 16).

In this study, we synthesized hydrophilic Ir(III) complexes conjugated with arginine peptides of different lengths (BTQ-R_n_ (n = 4, 8, 12, 16), Fig. 1) for in vivo vascular imaging. Ir(III) complex BTQphen, (btq)_2_Ir(phen-pipe) (btq = benzothienylquinoline, phen-pipe = 5-(1-piperazinyl)-1,10-phenanthroline), was selected as the phosphorescent core of BTQ-R_n_, although other phosphorescent metal complexes such as rare-earth metal complexes, Ru(II) complexes and metalloporphyrins might also be applicable as oligoarginine conjugates^24,25^. BTQ exhibits near-infrared emission, which is advantageous in terms of permeability to tissues, and can be excited at longer wavelengths than rare-earth metal complexes. The usefulness of BTQ-R_n_ as new vascular imaging probes was demonstrated by comparing the luminescence microscopic images of several organs and tumor tissues with those obtained using FITC-tomato lectin, often used as a vascular endothelial imaging reagent as mentioned earlier. In addition to conventional luminescence microscopic measurements, we used fluorescence lifetime imaging microscopy (FLIM) and phosphorescence lifetime imaging microscopy (PLIM) to reveal the utility of these newly-developed phosphorescent vascular probes.

## Results and discussion

### Photophysical properties and cellular uptake of BTQ-R_n_

**Figure 2:**
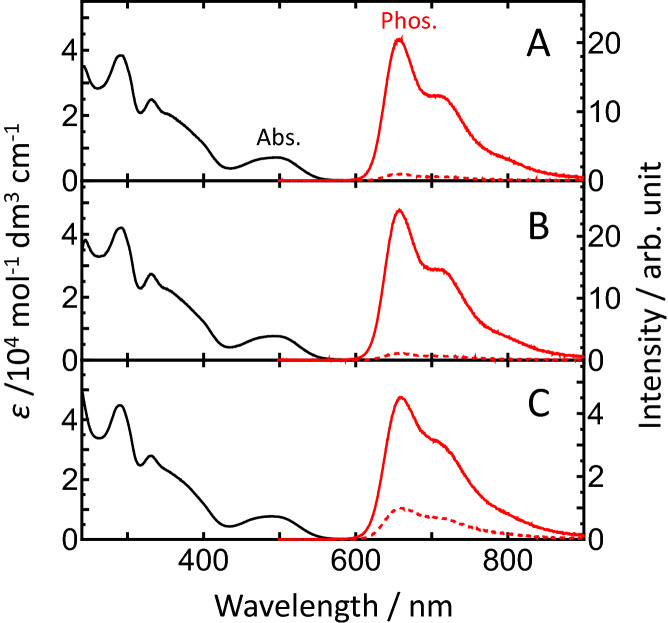
Absorption and phosphorescence spectra of BTQphen in MeCN (**A**), BTQ-R_12_ in MeCN (**B**), and BTQ-R_12_ in Tris–HCl buffer (pH 7.0) (**C**). These solvents contained 1% DMSO. (**A**) and (**B**) were measured at room temperature. Absorption spectra and phosphorescence spectra in (**C**) were measured at room temperature and 37 °C, respectively. The phosphorescence spectra were taken in both deaerated (solid line) and aerated (dashed line) solutions.

Ir(III) complexes BTQ-R_n_ (n = 4, 8, 12, 16) with different lengths of arginine peptides in the ligand (Fig. 1) were obtained by chemical synthesis, including solid-phase synthesis of the peptides. The synthetic details and characterization of BTQ-R_n_ are described in Supplementary Information. We first examined the spectral and photophysical properties of the synthesized BTQ-R_n_ (n = 4, 8, 12, 16) in MeCN and Tris–HCl buffer (pH 7.0) and compared them to the parent complex BTQphen. Figure 2A,B show the absorption and emission spectra of BTQphen and BTQ-R_12_ (a representative BTQ-R_n_) in MeCN. Both complexes exhibit very similar absorption and emission spectra. The first absorption band of BTQ-R_12,_ which has a molar absorption coefficient of 7600 mol^−1^ dm^3^ cm^−1^ at 496 nm, can be assigned to the singlet metal-to-ligand charge transfer (^1^MLCT) transition^26^. Emissions appear in the red to near-infrared region, with the maximum at around 660 nm, and show a very large Stokes shift. The emission decay profiles follow single-exponential functions, with lifetimes of 5.7 μs for BTQphen and 5.9 μs for BTQ-R_12_ in deaerated MeCN, and 0.28 μs for BTQphen and BTQ-R_12_ in aerated MeCN (Table 1). The extremely long lifetimes in deaerated solution and significant quenching by dissolved oxygen indicate that emission is due to phosphorescence. The spectral properties of BTQphen and all the BTQ-R_n_ complexes are very similar (Figure S1), indicating that the oligoarginine moiety has little effect on the electronic properties of these complexes. Furthermore, as shown in Table 1, the phosphorescence quantum yields (Φ_p_^0^ and Φ_p_) and lifetimes (τ_p_^0^ and τ_p_) of BTQ-R_n_ in deaerated and aerated MeCN are very close to those of BTQphen. In aqueous solution, BTQphen forms aggregates due to its extremely low water solubility whereas BTQ-R_n_ shows high solubility in water and has absorption and emission spectra similar to those in MeCN (Fig. 2C and Figure S2). The phosphorescence decay profiles of BTQ-R_n_ in aerated and deaerated Tris–HCl buffer (pH 7.0) (Figure S3) follow single-exponential decay functions, supporting the absence of aggregate formation in water. The Φ_p_^0^ and τ_p_^0^ values of BTQ-R_n_ in buffer are reduced compared to those in MeCN (Table 1), whereas Φ_p_ and τ_p_ are increased because the solubility of oxygen in water is significantly lower than that in MeCN^27^.

**Table 1:** Phosphorescence quantum yield and lifetime of BTQphen and BTQ-R_n_ (n = 4, 8, 12, 16) in MeCN and Tris–HCl buffer (pH 7.0).

Compound	$\Phi_{{\text{p}}}^{0}$^a^	$\Phi_{{\text{p}}}$^a^	$\tau_{{\text{p}}}^{0}$*/*µs^b^	$\tau_{{\text{p}}}$*/*µs^b^
BTQphen	0.32	0.015	5.7	0.28
BTQ-R_4_	0.30 (0.23)^a^	0.013 (0.061)	5.9 (3.5)	0.28 (0.70)
BTQ-R_8_	0.30 (0.20)	0.012 (0.046)	5.7 (3.5)	0.28 (0.74)
BTQ-R_12_	0.29 (0.20)	0.016 (0.051)	5.9 (3.5)	0.28 (0.73)
BTQ-R_16_	0.29 (0.20)	0.017 (0.050)	5.8 (3.5)	0.30 (0.74)

^a^At room temperature. ^b^At 37 °C. ^c^In parentheses are in Tris–HCl buffer (pH 7.0).

$\phi_{{\text{p}}}^{0}$ and $\tau_{{\text{p}}}^{0}$ denote the phosphorescence quantum yield and lifetime taken for deaerated solutions, $\phi_{{\text{p}}}$ and $\tau_{{\text{p}}}$ stands for the phosphorescence quantum yield and lifetime taken for aerated solutions.

We then investigated interactions of BTQ-R_n_ complexes with albumin by examining their phosphorescence decay properties in fetal bovine serum (FBS). The binding of Ir(III) complexes to albumin prolongs the emission lifetime as compared to in the absence of albumin because quenching by dissolved O_2_ is suppressed by the surrounding amino acid residues^28^. The phosphorescence decay curves of BTQ-R_n_ in FBS deviated from single-exponential decay and could be analyzed by double-exponential decay functions with lifetimes of 1.4–1.7 μs and 4.0–4.4 μs at 37 °C (Figure S4, Table S1). Both components give much longer lifetimes in FBS than in water (0.70–0.74 μs). BTQphen dissolved in FBS showed a phosphorescence decay profile similar to those of BTQ-R_n_, indicating that BTQphen was incorporated into albumin. These results suggest that the hydrophobic BTQ moiety of BTQ-R_n_ interacts with a binding site on albumin, with BTQ-R_n_ thus binding to two different domains in albumin. Vascular imaging in mice typically involves administering probe molecules intravenously and thus BTQ-R_n_ is expected to be transported in the blood while bound to albumin.

Oligoarginine is a representative water-soluble cell-penetrating peptide and functions as a carrier for transporting membrane-impermeable molecules to target cells^29–31^. Hence, oligoarginine in the ligand may aid the uptake of BTQ-R_n_ into cells. Indeed, emission microscope images of mouse hepatocyte cells (AML12 cells) incubated with 2.0 μM BTQ-R_n_ for 2 h showed that BTQ-R_n_ is taken up into cells and probably accumulates in endosomes (Figure S5). The cytotoxicity of BTQ-R_12_ to AML12 cells was evaluated with the WST assay (incubation time: 24 h in the presence of BTQ-R_12_). No cytotoxicity was observed up to 10 μM (Figure S6), demonstrating that BTQ-R_n_ has relatively low cytotoxicity.

### In vivo imaging of mouse renal vessels using BTQ-R_n_

**Figure 3:**
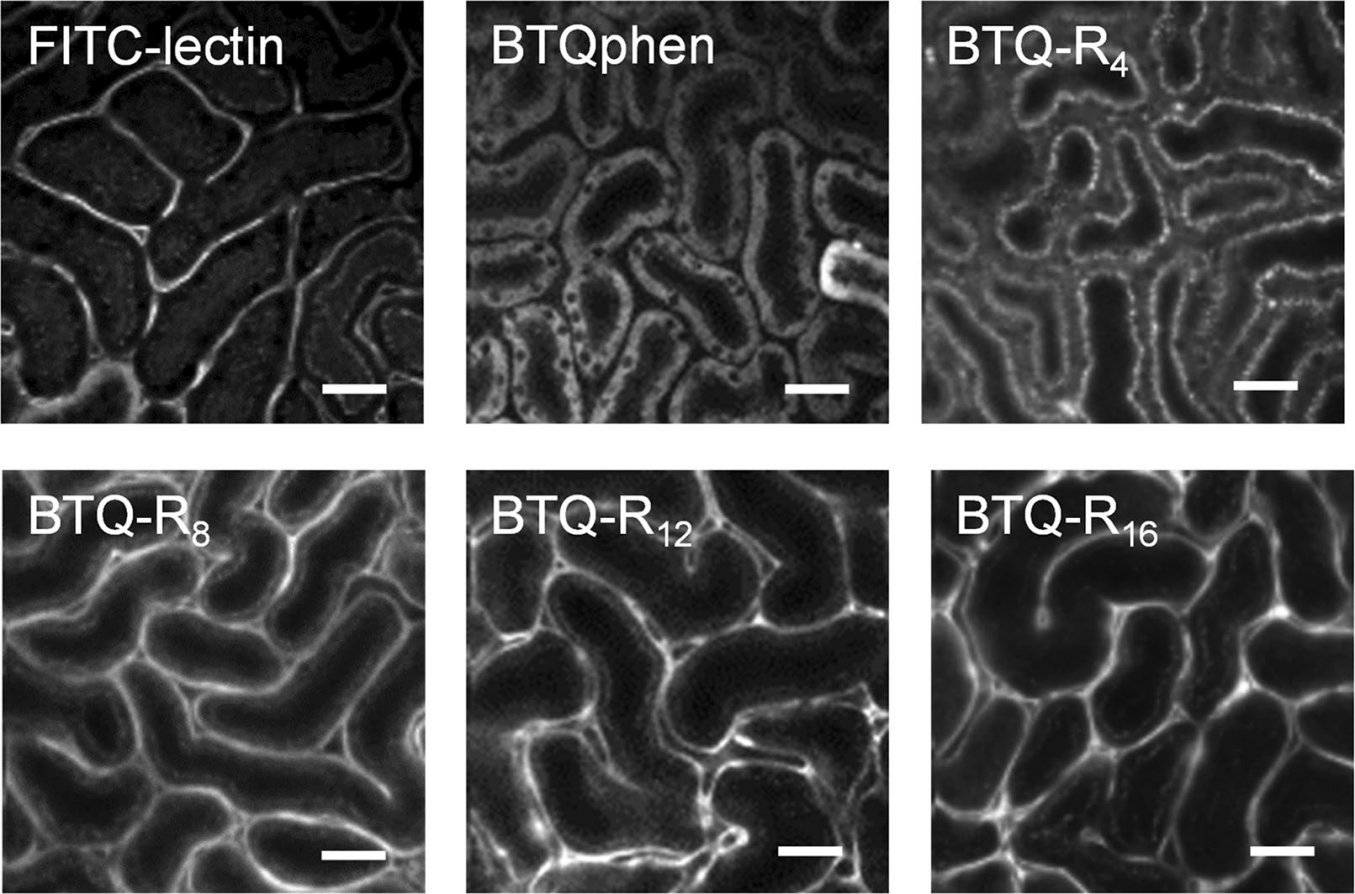
In vivo luminescence intensity images of mouse kidney after intravenous injection of FITC-lectin (1 mg/mL, 50 μL), BTQphen (100 nmol), and BTQ-R_n_ (n = 4, 8, 12, 16) (100 nmol). *λ*_exc_: 488 nm, *λ*_em_: > 590 nm (BTQ-R_12_), 510–560 nm (FITC-lectin). Scale bar 50 μm.

To evaluate the performance of BTQ-R_n_ as a vascular imaging probe, we intravenously administered BTQ-R_n_ or BTQphen (100 nmol) to anesthetized mice, then exposed the kidney by flank incision and obtained luminescence intensity images of the renal cortex. Similarly, FITC-lectin, known to bind to the vascular endothelium^16^, was administered to mice, and fluorescence images of the renal cortex were compared with the phosphorescence images obtained using BTQ-R_n_ or BTQphen (Fig. 3). All images taken approximately 1 h after probe administration clearly visualized the microstructure of renal cortex tissue^32^, but the localizations of the probes appeared to be different. Specifically, BTQphen localized in tubular cells rather than in capillaries and exhibited phosphorescence almost exclusively from inside the cells. This is consistent with the properties of cationic Ir(III) complexes with an *o*-phenanthroline ligand, i.e., being efficiently taken up into cells and accumulating in mitochondria^28,33^. BTQ-R_4_, which has the shortest arginine chain, also shows migration from the intravascular to the intracellular space, although the intracellular location appears to be different from that of BTQphen. In contrast, other BTQ-R_n_ compounds (n = 8, 12, 16) with longer arginine chains selectively image the endothelium of renal capillaries, similar to FITC-lectin. These results indicate that eight or more arginine residues are required to selectively stain the vascular endothelium using BTQ-R_n_. The emission images of BTQ-R_n_ (n = 8, 12, 16) in Fig. 3 were acquired using an excitation laser intensity equal to or lower than that used for FITC-lectin, although the detection wavelength was different (510–560 nm for FITC-lectin and > 590 nm for BTQ-R_n_). Even three hours after probe administration, clear capillary images were obtained with undiminished brightness (Figure S7A–D). This was in contrast to the short retention of fluorescent dextran with a molecular weight of 70 kDa that dissolve in blood plasma and circulate in the bloodstream (Figure S7E–H).

**Figure 4:**
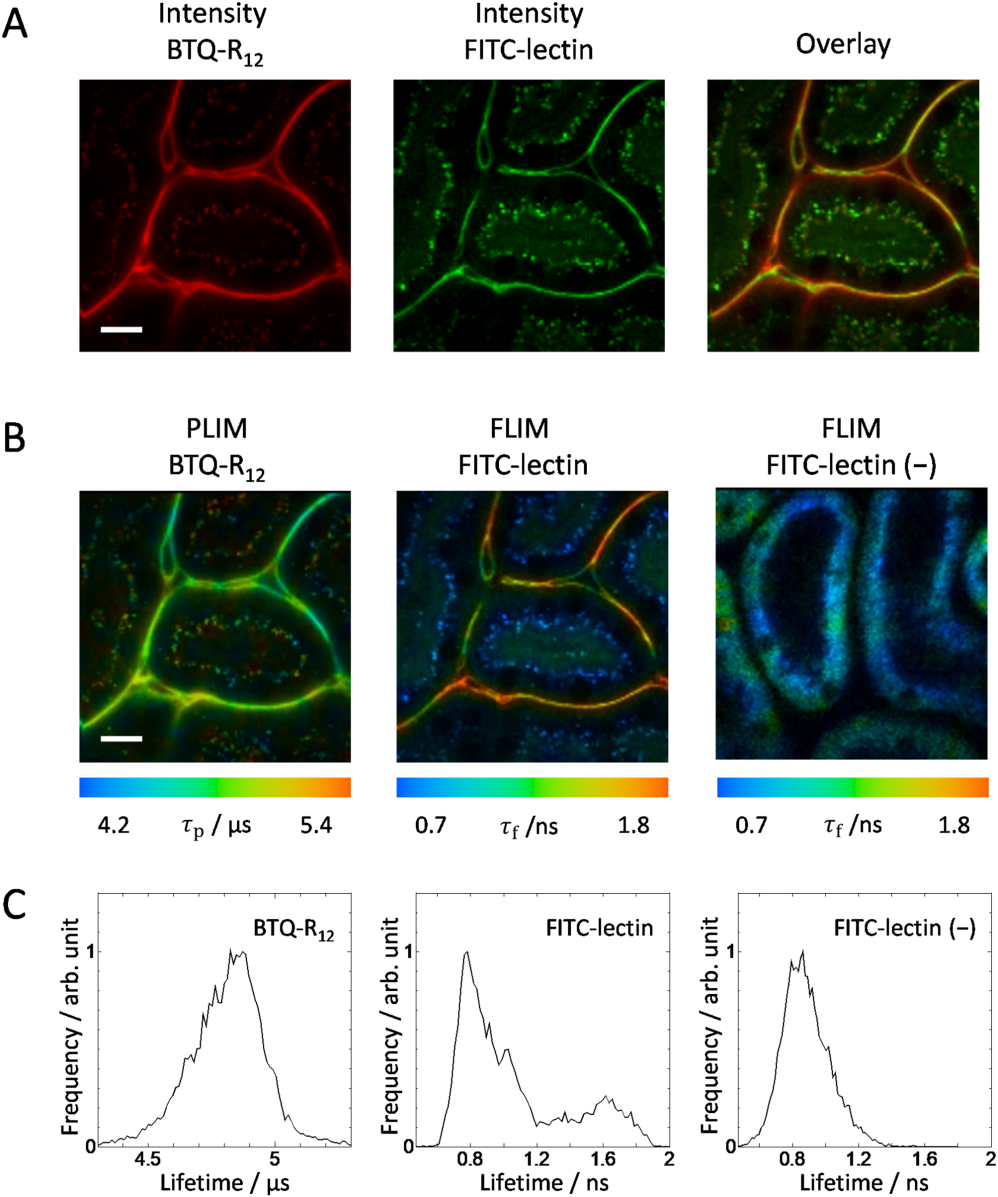
Luminescence intensity images (**A**) and PLIM or FLIM images (**B**) of mouse kidney at 1.5 h after intravenous injection of BTQ-R_12_ (100 nmol) and FITC-lectin (1 mg/mL, 50 μL). The right panel in (**A**) is the overlay image. A FLIM image of unstained renal cortex is shown in the right panel in (**B**). *λ*_exc_: 488 nm, *λ*_em_: > 590 nm (BTQ-R_12_), 510–560 nm (FITC-lectin). Scale bar 20 μm. (**C**) Distribution histograms of the luminescence lifetimes for the PLIM and FLIM images in (**B**).

The photostability of the vascular staining probe is an essential property for long-term observation of the target. We therefore investigated the photostability of BTQ-R_12_ bound to the vascular endothelium by taking emission intensity images of renal capillaries of a mouse administered with BTQ-R_12_ under continuous 488 nm laser pulse irradiation. The intensity images of renal capillaries were taken at every 50 frames until 500 frames (Figure S8A). During this irradiation time, the phosphorescence intensity of BTQ-R_12_ showed only a slight decrease (Figure S8B). In this study, the phosphorescence signals accumulated with 50 frames were usually averaged to obtain a single emission intensity image. Therefore, BTQ-R_12_ was found to have sufficient photostability to track changes in the vascular network in tissues. In addition, we measured the emission intensity image in the same region 1 h after measuring the 500 frames to reveal the photodamage to the tissue (Figure S8C). No significant change was observed in the emission image, including the morphology of the capillaries. This indicates that there is little photodamage to the tissue that can be caused by the singlet oxygen generated by the BTQ-R_12_ excitation. The reason why BTQ-R_12_ causes almost no photodamage to the tissue under these excitation conditions is considered to be related to the decrease in the phosphorescence quenching efficiency due to oxygen in the tissue; the phosphorescence lifetimes of BTQ-R_12_ in tissues (see Fig. 4) are significantly longer than those in aerated solutions (see Table 1), suggesting the much lower yield for singlet oxygen production in tissues^34^.

**Figure 5:**
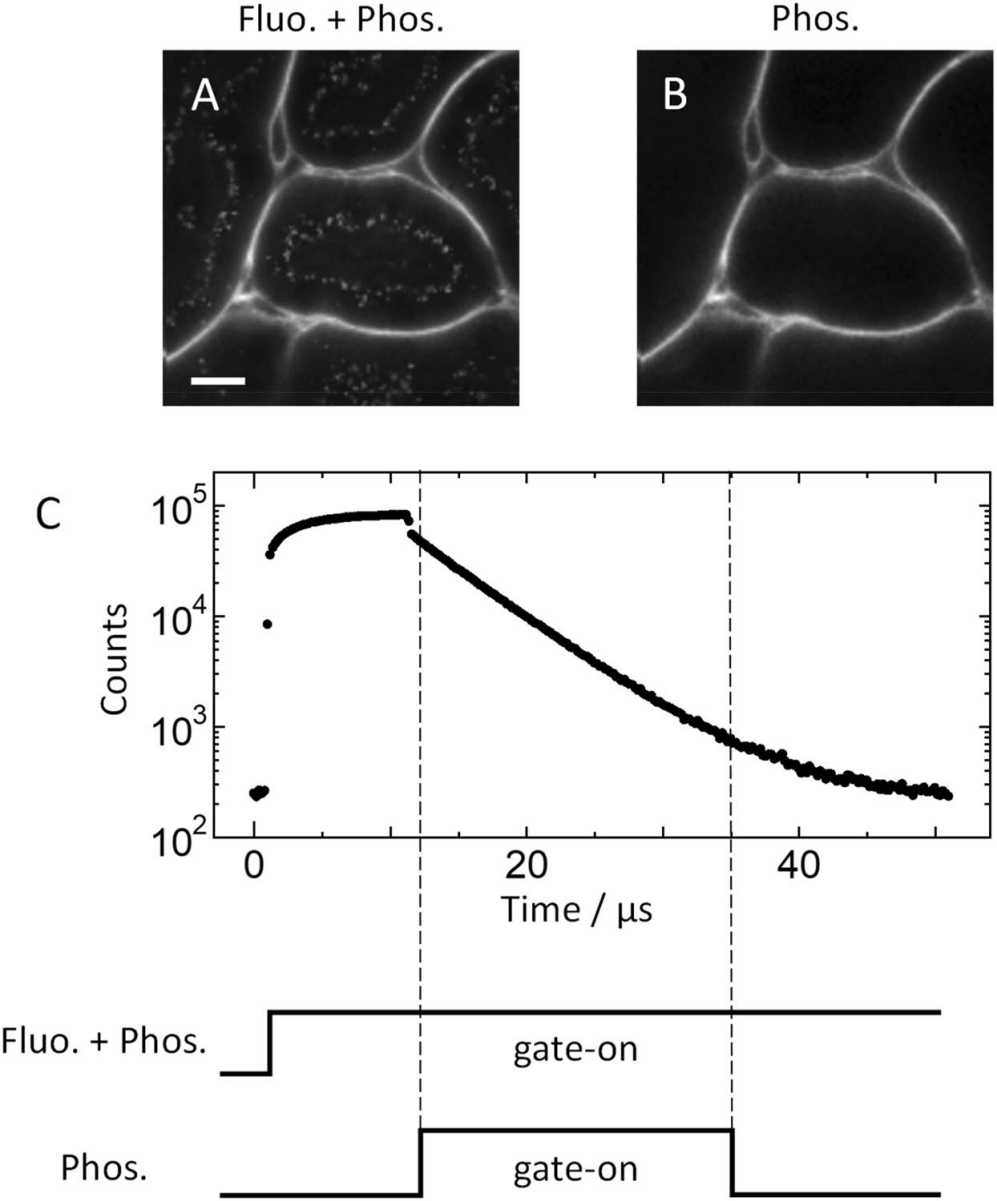
Elimination of autofluorescence from kidney using the time gating method. (**A**) Luminescence intensity image of BTQ-R_12_ including tissue autofluorescence and (**B**) its gated image. Scale bar 20 μm. (**C**) Luminescence decay curve of the total pixels in image (**A**). The schematic shows the time domains of (**A**) and (**B**). The dashed lines show the start and end of the time gate. Gate: 12–35 μs.

We used BTQ-R_12_ to further investigate the usefulness of BTQ-R_n_. First, we performed colocalization analyses to compare the stained areas of FITC-lectin and BTQ-R_12_ in the renal cortex (Fig. 4A). Emission intensity images of renal cortex obtained by simultaneous administration of BTQ-R_12_ and FITC-lectin are shown in the pseudo colors red and green, respectively, and their overlay image is shown on the right. The emission image obtained following BTQ-R_12_ administration (Fig. 4A, left) comprises both long-lived phosphorescence and short-lived fluorescence components. As can be seen from the overlay image, BTQ-R_12_ and FITC-lectin exhibit emission from the vascular endothelium, demonstrating similar localization within capillaries. This suggests that BTQ-R_12_, like FITC, specifically binds to endothelial cells in blood vessels. Figure 4A shows that emission originates not only from the capillaries but also inside the tubular cells and is observed as bright speckles. To clarify the origin of emission from the cells, we next obtained FLIM/PLIM images of renal cortex using BTQ-R_12_ and FITC-lectin (Fig. 4B). It should be noted here that the color scales for lifetime are significantly different between PLIM images obtained using BTQ-R_12_ and FLIM images obtained using FITC-lectin. The distribution histogram of the emission lifetime in the renal FLIM image obtained using FITC-lectin (Fig. 4C) indicates that the FLIM image obtained by staining with FITC-lectin comprises at least two different components with average lifetimes of around 0.8 ns and 1.6 ns. The short and long lifetime components can be assigned to autofluorescence by the renal cortex and the fluorescence of FITC-lectin, respectively, given that (1) the lifetime distribution of control images without FITC-lectin (Fig. 4C, right) approximate the distribution of the shorter lifetime component of FITC-lectin, and (2) the fluorescence lifetime (3.68 ns) of FITC-lectin in saline solution is longer than that of the shorter lifetime component. It is important to note here that it is difficult to remove the contribution of autofluorescence in FITC-lectin-based images because of the close proximity of the emission wavelengths of autofluorescence and FITC-lectin, and it is difficult to remove autofluorescence with a filter. In contrast, with BTQ-R_12_, the autofluorescence component can be removed due to the large difference in lifetime between autofluorescence and BTQ-R_12_ phosphorescence (Fig. 5). Figure 5C shows the accumulation of phosphorescence due to repeated irradiation with pulsed laser light and the subsequent phosphorescence decay profile when the laser is turned off. During the accumulation of phosphorescence, the fluorescence decays rapidly after each pulsed excitation. The image shown in Fig. 5A was obtained by observing the emission over the entire time domain, and that in Fig. 5B is a gated image in which only the phosphorescence component was observed after the laser pulse was turned off. As can be seen from Fig. 5B, autofluorescence is absent and only the capillaries of the renal cortex are clearly imaged. The removal of autofluorescence by using a time-gated emission detection method is an important advantage of phosphorescent probes^35^.

### Visualization of blood vessels in normal and pathological tissues

**Figure 6:**
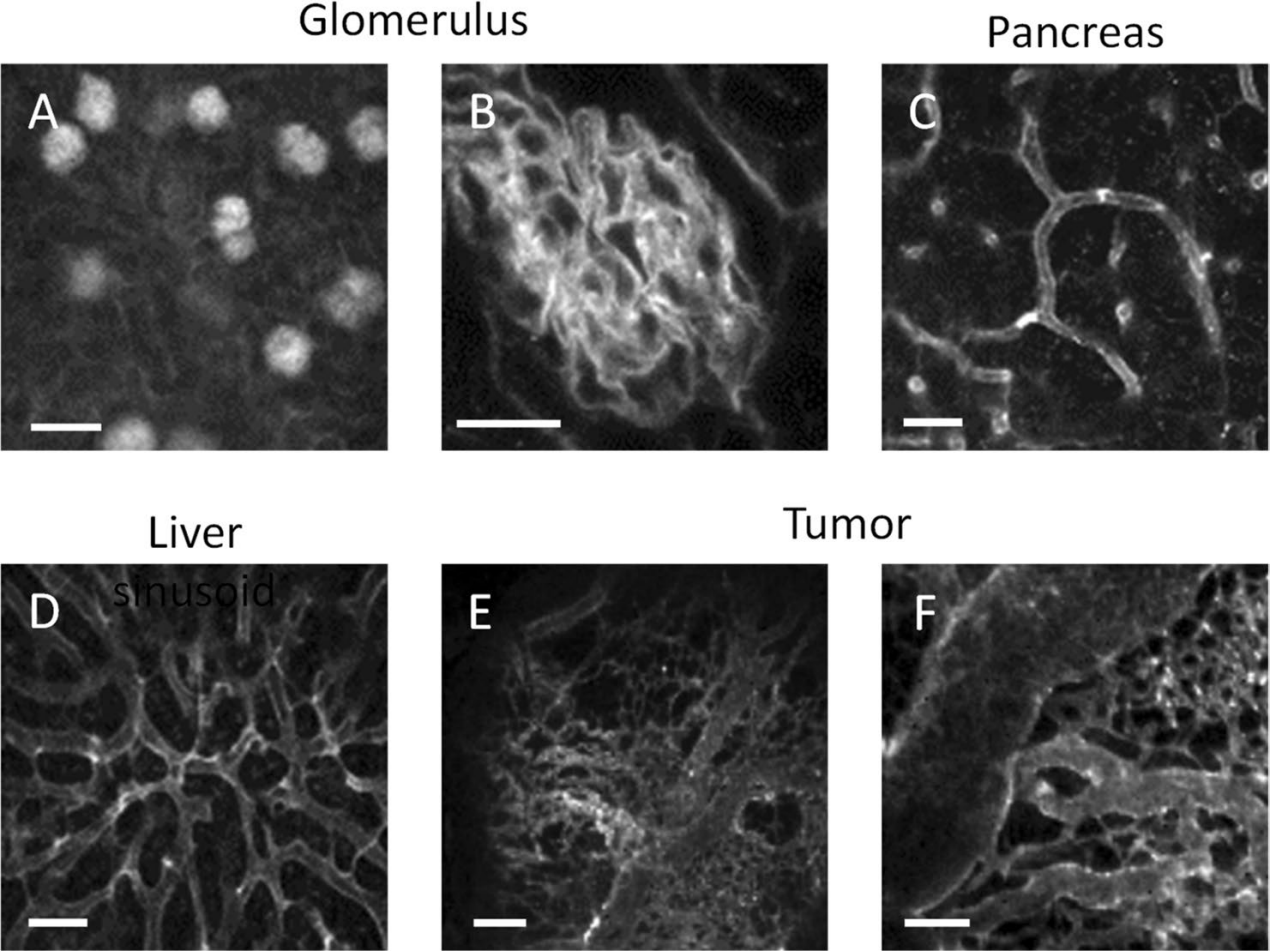
Phosphorescence intensity images of kidney (**A**), glomerulus (**B**), liver sinusoids (**C**), and pancreas (**D**) in living mice administrated BTQ-R_12_ (100 nmol) via the tail vein under anesthesia. (**E**,**F**) Phosphorescence intensity images of tumor vascular networks. *λ*_exc_: 488 nm, *λ*_em_: > 590 nm. Scale bar: 100 μm in (**A**), 20 μm in (**B**), 50 μm in (**C**), 50 μm in (**D**), 500 μm in (**E**), 100 μm in (**F**).

The confocal microscope images obtained using our PLIM system are limited to a depth of approximately 30 μm from the renal surface. Glomerular images could therefore not be obtained and so we performed PLIM measurements on mouse kidney sections following the administration of BTQ-R_12_ (Fig. 6A,B). As can be seen from the high magnification image of a kidney section in Fig. 6B, BTQ-R_12_ can be used to image the fine vascular network of the glomeruli. The administration of BTQ-R_12_ allows in vivo and ex vivo imaging of the microvasculature in a variety of organs and tissues, including pancreas, liver, small intestine, adrenal gland, hypodermal tissue, bone marrow, lung and heart (Fig. 6C,D and Figure S9).

Next, we attempted in vivo vascular imaging of tumor tissues by intravenously administrating BTQ-R_12_ to tumor-bearing mice. Figure 6E,F show phosphorescence intensity images of a solid tumor that grew for 2 weeks after transplanting HCT-116 cells (5 × 10^6^ cells) subcutaneously into the lower thigh of mice. The images were taken after removing the skin covering the tumor, placing the exposed tumor on a thin glass plate on a microscope stage, and irradiating the surface with excitation laser light from below. The phosphorescence intensity images of different regions of the same tumor at different magnifications show large blood vessels and a large number of small neovasculatures branching from them. It is clear from Fig. 6C,D that the vascular structure of the tumor tissue is extremely heterogeneous, in contrast to the structure of the sinusoids in the liver, which are arranged in interconnecting polygonal networks.

**Figure 7:**
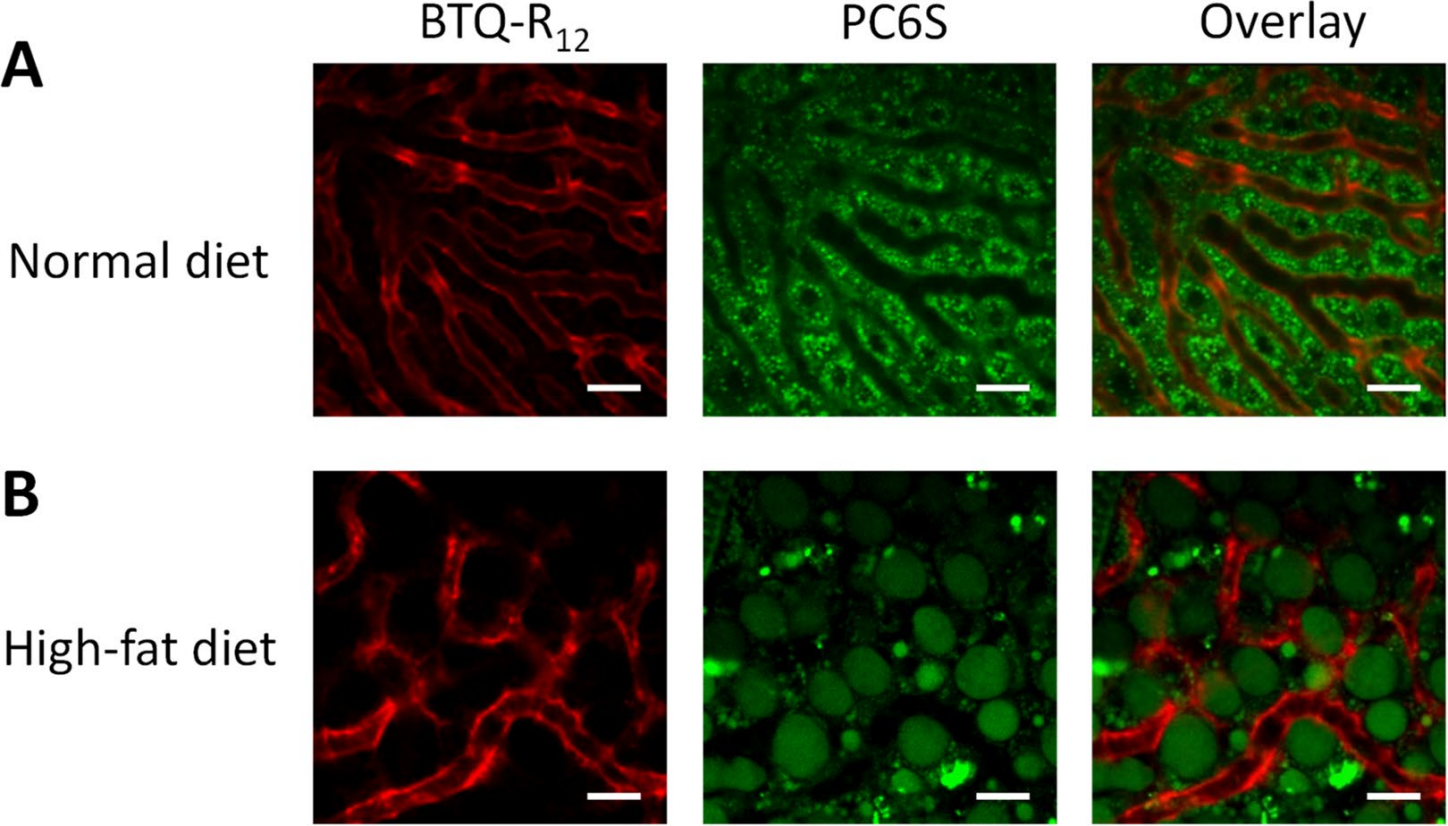
Phosphorescence and fluorescence intensity images of sinusoids (BTQ-R_12_, 100 nmol) and hepatic lipid droplets (PC6S, 50 nmol) in the liver of mice fed a normal diet (**A**) or high-fat diet (**B**) for 2 weeks. *λ*_exc_: 488 nm, *λ*_em_: > 620 nm (BTQ-R_12_), 510–560 nm (PC6S). Scale bar: 50 μm.

The liver is a highly vascular organ and receives approximately 30% of resting cardiac output. In the hepatic portal system, the liver receives blood both from the hepatic portal vein and the hepatic arteries, and the blood mixes in the sinusoids. The sinusoids are uniformly distributed throughout the entire liver volume and constitute the hepatic microcirculation. The onset and progression of liver diseases is expected to cause changes in the network structure of the sinusoid^36^. Here, we used BTQ-R_12_ to obtain luminescence microscope images of the liver of mice fed a high-fat diet for 2 weeks and compared the organization of the sinusoid network with that of healthy mice. The mice fed a high-fat diet for 2 weeks are expected to have fatty livers due to lipid accumulation. Lipid droplets (LDs) are formed as diseases such as nonalcoholic fatty liver disease (NAFLD) and nonalcoholic steatohepatitis (NASH) progress^37–39^ and thus we co-administered the green fluorescent LD-specific probe PC6S^40^ to the mice. Figure 7 shows phosphorescence and fluorescence intensity images of liver sinusoids of living mice fed a normal diet and a high-fat diet. The LD-specific probe PC6S accumulates in the endoplasmic reticulum (ER) and more selectively in LDs in cells^40^. Figure 7A shows that PC6S is taken up into hepatocytes and likely accumulates in LDs and the ER. Overlay of the sinusoid image (red) obtained using BTQ-R_12_ and the hepatocytes image (green) obtained using PC6S clearly visualizes the entire microstructure of the hepatic tissue. In contrast, many LDs are apparent in the liver of mice fed a high-fat diet, and as a result the sinusoidal structure is significantly distorted (Fig. 7B).

These results demonstrate that BTQ-R_12_ is useful for selectively staining the vascular endothelium of various organs as well as tumors, and for investigating their vascular structure and dynamics in vivo. Judging from the extremely long phosphorescence lifetime of BTQ-R_12_ within kidney capillaries (Fig. 4) compared to that in aerated solution, BTQ-R_12_ is likely taken up by vascular endothelial cells where its interaction with oxygen is suppressed, although BTQ-R_12_ may be bound to the vascular endothelium while bound to albumin. Further research is required to clarify how BTQ-R_12_ binds to the vascular endothelium following intravenous administration to mice.

## Conclusions

The deep-red phosphorescent vascular imaging probes BTQ-R_n_ (n = 8, 12, 16), in which a Ir(III) complex is connected with an oligoarginine peptide, were designed and synthesized by conventional chemical synthesis. We demonstrated that the blood vessels of various tissues and tumors in mice can be visualized using a confocal luminescence microscope following the intravenous administration of BTQ-R_12_. The long luminescence lifetime of BTQ-R_12_ coupled with gated measurements allowed visualization of the vascular structure of organs without interference from autofluorescence. BTQ-R_12_ allowed long-term in vivo vascular network imaging because it selectively binds to vascular endothelial cells. Furthermore, dual color imaging of normal and fatty liver in living mice using BTQ-R_12_ and the green fluorescent lipid droplet probe PC6S showed distortion of the sinusoid network in mice fed a high-fat diet for 2 weeks. These results demonstrated that BTQ-R_12_, combined with a confocal luminescence microscope, can be useful for imaging blood vessels in normal and pathological tissues.

## Methods

### Materials

BTQphen and BTQ-R_n_ (n = 4, 8, 12, 16) were synthesized and identified according to the methods described in Supplementary Information. MeCN (fluorometric grade, Kanto Chemical), tris(hydroxymethyl)aminomethane (Tris, Sigma-Aldrich), FITC-dextran (average molecular weight; 70 kDa, Sigma-Aldrich) and FBS (Thermo Fisher Scientific) were used as received. FITC-lectin (Vector Laboratories) was purchased from Funakoshi.

### Absorption and emission spectra

Absorption and emission spectra were recorded on a UV–Vis spectrophotometer (Ubest V-550, JASCO) and a photonic multichannel analyzer PMA-12 (C11027-01, Hamamatsu Photonics) equipped with a monochromatized Xe arc lamp, respectively. The emission spectra were fully corrected for spectral sensitivity.

### Phosphorescence lifetimes and phosphorescence quantum yields

Phosphorescence lifetimes were measured with a time-correlated single-photon counting fluorimeter (Quantaurus-Tau C11367G, Hamamatsu Photonics). A laser diode (M12488-35, Hamamatsu Photonics; 481 nm, pulse width 70 ps) was used as the excitation light source. The temperature of the sample solution was controlled by circulating water through a jacket cuvette holder from a temperature controlled bath (RTE7, Neslab). Phosphorescence quantum yields were determined on an absolute photoluminescence quantum yield measurement system (C9920-02, Hamamatsu Photonics) consisting of a Xe arc lamp, a monochromator, an integrating sphere, and a multichannel detector^41^.

### Cell lines and culture

Alpha mouse liver 12 (AML12) cells were kindly gifted by Prof. T. Inagaki of the Laboratory of Epigenetics and Metabolism, IMCR, Gunma University. Human colorectal carcinoma (HCT-116) cells were purchased from the American Type Culture Collection. AML12 cells were cultured in Dulbecco’s modified Eagle’s medium: nutrient mixture F-12 (DMEM/F12, Thermo Fisher Scientific). HCT-116 cells were cultured in McCoy’s 5A medium. These media were supplemented with 10% fetal bovine serum (FBS, Thermo Fisher Scientific), penicillin (50 units/mL, Thermo Fisher Scientific) and streptomycin (50 μg/mL, Thermo Fisher Scientific). All cells were grown at 37 °C under a 5% CO_2_ atmosphere.

### Cell viability assay

AML12 cells (2 × 10^4^ cells/well) were seeded into a 96-well flat bottom plate (Greiner) and allowed to adhere for 24 h. A stock solution of BTQ-R_12_ (1 mM) in DMSO was diluted with DMEM/F12 containing 10% fetal bovine serum, penicillin (50 units/mL) and streptomycin (50 μg/mL). The cells were incubated with the probe for 24 h at 37 °C under a 5% CO_2_ atmosphere. The medium was removed and the cells were washed gently with DMEM/F12, then the medium was replaced with DMEM/F12 without phenol red. Cell Counting Kit-8 reagent (CCK-8, Dojindo) was added to each well and incubation was continued for 2 h. The absorbance at 450 nm of each well referenced at 650 nm was recorded using a microplate reader (Infinite 200 PRO, TECAN). Cell viability (% of control) was evaluated as (A_sample _− A_blank_)/(A_control _− A_blank_) × 100, where A_sample_ is the absorbance of cells exposed to the probe, A_control_ is the absorbance of cells without probe, and A_blank_ is the absorbance of wells without cells^28^.

### Animal handling and ethics

All protocols for animal experiments were approved by the Ethical Committee on Animal Experiments of Gunma University (18-018), and all animal experiments were conducted under the institutional guidelines. All experiments were performed in compliance with the ARRIVE guidelines. Seven- to nine-week-old Balb/c male mice (CLEA Japan) were used in this study. Tumor transplants were established in 7-week-old male athymic Balb/c nude mice (nu/nu) (CLEA Japan) by subcutaneous injection of a suspension of HCT-116 cells [5 × 10^6^ cells in 150 μL PBS:Matrigel (1:1 v/v)]. Experiments with tumor-bearing mice were performed 2 weeks after the injection of tumor cells. Fatty liver model mice were generated by feeding 6-week-old Balb/c male mice (CLEA Japan) with a choline-deficient, l-amino acid-defined, high-fat diet (CDAHFD, A06071302, Research Diets) consisting of 60 kcal% fat and 0.1% methionine for 2 weeks^42^.

### In vivo phosphorescence intensity and lifetime imaging

A stock solution of BTQphen (1 mM) was prepared by dissolving in saline:DMSO (9:1 v/v). Stock solutions of BTQ-R_n_ (1 mM) and FITC-dextran (1 mg/mL) were prepared by dissolving in saline. A stock solution of PC6S (0.5 mM) was prepared by dissolving in saline:DMSO (9:1 v/v) containing 10 wt% BSA. Mice were anaesthetized by intraperitoneal injection of mixed anesthetic (ketamine: 100 mg/kg, xylazine: 10 mg/kg in saline). Stock solutions of BTQphen (1 mM, 100 μL), BTQ-R_n_ (1 mM, 100 μL), FITC-lectin (1 mg/mL, 50 μL), and FITC-dextran (1 mg/mL, 100 μL) were injected into the tail vein under anesthesia. For dual color imaging experiments, a stock solution of PC6S (0.5 mM, 100 μL) was injected into the tail vein under anesthesia, and 10 min later BTQ-R_12_ solution (1 mM, 100 μL) was injected into the tail vein. The kidney or other tissue was exposed by flank or median incision, then the mouse was turned with its exposed tissue on a cover glass chamber (Iwaki). Luminescence intensity and lifetime images were recorded on an inverted fluorescence microscope (IX73, Olympus) equipped with a confocal scanning system (DCS-120, Becker & Hickl). The excitation light source was a picosecond diode laser (BDL-SMC, wavelength: 488 nm, pulse width: 40–90 ps, repetition rate: 20 MHz, Becker & Hickl). Emission was detected with a hybrid detector module (HPM-100-40, Becker & Hickl)^32^. Emission decay curves were analyzed using SPCImage data analysis software (Becker & Hickl) to obtain emission intensity, lifetime images, and gated image.

## Supplementary Information

Supplementary Information.
