# Supplementary material for: Phosphorescent Ir(III) complexes conjugated with oligoarginine peptides serve as optical probes for in vivo microvascular imaging
Source: Sci Rep. 2021 Feb 26;11:4733. doi: 10.1038/s41598-021-84115-x (PMC7910296; doi:10.1038/s41598-021-84115-x)
Supplement: Supplementary file 1 — Supplementary Information. [file 41598_2021_84115_MOESM1_ESM.pdf]

Supplementary Information

Phosphorescent Ir(III) complexes conjugated with oligoarginine peptides serve as optical probes for in vivo microvascular imaging

Mami Yasukagawa, Aya Shimada, Shuichi Shiozaki, Seiji Tobita and Toshitada Yoshihara*

Department of Chemistry and Chemical Biology, Graduate School of Science and Technology, Gunma University, Kiryu 376-8515, Japan.

*To whom correspondence should be addressed.

E-mail: yoshihara@gunma-u.ac.jp

This file includes:

Synthesis of BTQphen, BTQ-R_4, BTQ-R_8, BTQ-R_{12}, and BTQ-R_{16}

Fig. S1 to S9

Table S1

Materials

All reagents and solvents were purchased from BeadTech, Watanabe Chemical Industries, Peptide Institute, Tokyo Chemical Industries, Wako Pure Chemical Industries, or Kanto Chemical, and were used without further purification. ^{1}H-NMR spectra were recorded with a JNM-ECS400 (JEOL) at 400 MHz. ^{1}H-NMR chemical shifts were referenced to tetramethylsilane. ESI-MS and MALDI-TOF-MS measurements were carried out on an API 2000 (Applied Biosystems) and an AXIMA performance (Shimadzu) mass spectrophotometers.

BTQphen

BTQphen was synthesized according to the literature[1]. 2-(Benzo[*b*]thiophen-2-yl)quinoline (1.90 g, 7.2 mmol) and $IrCl_3 \cdot 3H_2O$ (1.24 g, 3.5 mmol) were dissolved in 2-ethoxyethanol (90 mL) and distilled water (35 mL) and then the solution was heated at reflux for 15 h. After cooling, the precipitate formed was filtered to give a chloro-briged dimer of BTQ washed thoroughly with methanol and *n*-hexane. *tert*-Butyl 4-(1,10-phenanthrolin-5-yl)piperazine-1-carboxylate (397 mg, 1.1 mmol) and chloro-bridged dimer of BTQ (752 mg, 0.5 mmol,) were dissolved in tetrahydrofuran (50 mL) and methanol (50 mL), then the solution was refluxed at 6 h. After cooling, KPF_6 (376 mg, 2.0 mmol) was added to the solution and stirred at 1h. The solution was evaporated to dryness under reduced pressure. The crude product was purified by aminopropyl-functionalized silica-gel column chromatography using chloroform as eluent. The product (BTQphen-pipe-Boc) was obtained as red powder (731 mg, 0.60 mol, 60%).

The deprotection cocktail (TFA/Triisopropylsilane (TIPS) /H_2O 95:2.5:2.5, 1 mL) was added to BTQphen-pipe-Boc (516 mg, 0.42 mmol) in a centrifuge tube. After 2 h at room

temperature, a red solid was precipitated by addition of the cool diethyl ether. The precipitate was centrifuged (3500 rpm, 5 min), then diethyl ether was removed by decantation. This operation was repeated twice. The product (BTQphen) was obtained as red powder (444 mg, 0.36 mol, 85%).

^{1}H NMR (400 MHz, DMSO-D6) δ 8.92 (br, 2H), 8.77 (d, J = 8.5 Hz, 1H), 8.68 (d, J = 5.3 Hz, 1H), 8.56 (d, J = 8.5 Hz, 1H), 8.52-8.41 (m, 3H), 8.20 (q, J = 8.6 Hz, 2H), 8.08 (dd, J = 8.5, 5.3 Hz, 1H), 8.05-7.92 (m, 3H), 7.76 (dd, J = 11.3, 8.1 Hz, 2H), 7.59 (s, 1H), 7.20-7.10 (m, 4H), 6.93 (d, J = 8.9 Hz, 1H), 6.78-6.60 (m, 5H), 6.21 (dd, J = 13.3, 8.2 Hz, 2H), 3.53 (br, 4H), 3.13 (br, 4H), ESI-MS (positive) : calcd. for $C_{50}H_{36}IrN_6S_2$ (M^+): 977.21, found: 977.2.

Boc-[R(Pbf)]$_n$-OH (n = 4, 8, 12, 16)

Peptides were synthesized by using a fully automated microwave peptide synthesizer (Biotage Initiator+ Alstra) based on solid-phase synthesis. H-R(Pbf)-2-Chlorotrityl resin (BeadTech, 0.38 mmol/g loading) was used as a solid support. For the coupling of an amino acid, 0.5 M Fmoc-[R(Pbf)]-OH in DMF, 0.5 M Boc-[R(Pbf)]-OH in DMF, 0.6 M HBTU in DMF, 0.5 M HOBt in DMF and 2.0 M *N*,*N*-diisopropylethylamine (DIPEA) in NMP were added into reaction vial (3 eq., 3 eq., 3 eq. and 6 eq. respectively). The reaction mixture was stirred for 1 h at room temperature. Deprotection of Fmoc group was performed in 2% DBU in DMF for 5 min at room temperature. For complete deprotection, the reaction was carried out again under the same conditions for 10 minutes. After the peptide elongation, for cleaving peptide from resin, 1% TFA in DCM (10 mL) was added into the vial, and the solution was stirred for 90 min at room temperature. The reaction mixture was filtered and filtrate evaporated under vacuum. The crude peptide was

dissolved in acetonitrile, and distilled water was added. This peptide solutions were lyophilized.

BTQ-R_4

Boc-[R(Pbf)]$_4$-OH (116 mg, 0.065 mmol), BTQphen (63 mg, 0.051 mmol), and HATU (41 mg, 0.10 mmol) were dissolved in anhydrous DMF (1 mL), then DIPEA (0.17 mL, 2.0 mmol) was added. This solution was stirred at room temperature for 24 h under N_2 gas. The distilled water was added into the reaction mixture. The precipitate was centrifuged (3500 rpm, 5 min), then water was removed by decantation. This operation was repeated twice. Any remaining water was removed from vessel by vacuum freezing and the product (BTQ-[R(Pbf)]$_4$-Boc) was obtained as red powder. The deprotection cocktail (TFA/TIPS/H_2O 95:2.5:2.5) (1 mL) was added to BTQ-[R(Pbf)]$_4$-Boc (279 mg, 0.10 mmol) in a centrifuge tube. After 2 h at room temperature, an orange solid was precipitated by addition of the cool diethyl ether. The precipitate was centrifuged (3500 rpm, 5 min), then diethyl ether was removed by decantation. This operation was repeated twice. The diethyl ether was removed by decantation, and then the orange solid was dried in a vacuum desiccator. The crude product was obtained as orange powder (82 mg, 40%). A part of the crude product was purified by purified by using SNAP Ultra C18 (Biotage); eluent, 30-100% acetonitrile aq. containing 0.1% TFA. The main peak fraction was collected and purified water was added. The aqueous solution was lyophilized to afford the product (BTQ-R_4) as orange powder.

ESI-MS (positive): calcd. for $C_{74}H_{84}IrN_{22}O_4S_2$ ($[M+2H]^{2+}$): 534.8, found: 534.8

MALDI-TOF MS: calcd. for $C_{74}H_{84}IrN_{22}O_4S_2^+$: 1601.9, found: 1601.2

BTQ-R_8

Boc-[R(Pbf)]$_4$-OH (209 mg, 0.062 mmol), BTQphen (62 mg, 0.050 mmol), and HATU (40 mg, 0.11 mmol) were dissolved in anhydrous DMF (1 mL), then DIPEA (0.17 mL, 1.0 mmol) was added. This solution was stirred at room temperature for 24 h under N_2 gas. The distilled water was added into the reaction mixture. The precipitate was centrifuged (3500 rpm, 5 min), then water was removed by decantation. This operation was repeated twice. Any remaining water was removed from vessel by vacuum freezing and the product (BTQ-[R(Pbf)]$_8$-Boc) was obtained as red powder. The deprotection cocktail (TFA/TIPS/H_2O 95:2.5:2.5) (1 mL) was added to BTQ-[R(Pbf)]$_8$-Boc (110 mg, 0.026 mmol) in a centrifuge tube. After 2 h at room temperature, an orange solid was precipitated by addition of the cool diethyl ether. The precipitate was centrifuged (3500 rpm, 5 min), then diethyl ether was removed by decantation. This operation was repeated twice. The diethyl ether was removed by decantation, and then the orange solid was dried in a vacuum desiccator. The crude product was obtained as orange powder (72 mg, 85%). A part of the crude product was purified by purified by using reversed phase flash chromatography (Biotage) and the following condition: column, SNAP Ultra C18 (Biotage); eluent, 30-100% acetonitrile aq. containing 0.1% TFA. The main peak fraction was collected and purified water was added. The aqueous solution was lyophilized to afford the product (BTQ-R_4) as orange powder.

ESI-MS (positive): calcd. for $C_{98}H_{132}IrN_{38}O_8S_2$ ($[M+3H]^{3+}$): 743.0, found: 742.6

MALDI-TOF MS: calcd. for $C_{98}H_{132}IrN_{38}O_8S_2^+$: 2226.6, found: 2224.8

BTQ-R_{12}

Boc-[R(Pbf)]$_{12}$-OH（321 mg，0.064 mmol）, BTQphen (62 mg，0.050 mmol), and HATU (40 mg，0.11 mmol) were dissolved in anhydrous DMF (1 mL), then DIPEA (0.17 mL, 1.0 mmol) was added. This solution was stirred at room temperature for 24 h under N_2 gas. The distilled water was added into the reaction mixture. The precipitate was centrifuged (3500 rpm, 5 min), then water was removed by decantation. This operation was repeated twice. Any remaining water was removed from vessel by vacuum freezing and the product (BTQ-[R(Pbf)]$_{12}$-Boc) was obtained as red powder. The deprotection cocktail (TFA/TIPS/H_2O 95:2.5:2.5) (1 mL) was added to BTQ-[R(Pbf)]$_{12}$-Boc（151 mg，0.026 mmol）in a centrifuge tube. After 2 h at room temperature, an orange solid was precipitated by addition of the cool diethyl ether. The precipitate was centrifuged (3500 rpm, 5 min), then diethyl ether was removed by decantation. This operation was repeated twice. The diethyl ether was removed by decantation, and then the orange solid was dried in a vacuum desiccator. The crude product was obtained as orange powder (93 mg, 81%). A part of the crude product was purified by purified by using reversed phase flash chromatography (Biotage) and the following condition: column, SNAP Ultra C18 (Biotage); eluent, 30-100% acetonitrile aq. containing 0.1% TFA. The main peak fraction was collected and purified water was added. The aqueous solution was lyophilized to afford the product (BTQ-R$_{12}$) as orange powder.

ESI-MS (positive): calcd. for $C_{122}H_{180}IrN_{54}O_{12}S_2$ ($[M+4H]^{4+}$): 713.6, found: 713.

MALDI-TOF MS: calcd. for $C_{122}H_{180}IrN_{54}O_{12}S_2^{+}$: 2851.4, found: 2846.8

BTQ-R$_{16}$

Boc-[R(Pbf)]$_{16}$-OH（183 mg，0.028 mmol）, BTQphen (37 mg，0.030 mmol), and HATU (35 mg，0.09 mmol) were dissolved in anhydrous DMF (1 mL), then DIPEA (0.17 mL, 1.0

mmol) was added. This solution was stirred at room temperature for 24 h under N_2 gas. The distilled water was added into the reaction mixture. The precipitate was centrifuged (3500 rpm, 5 min), then water was removed by decantation. This operation was repeated twice. Any remaining water was removed from vessel by vacuum freezing and the product (BTQ-[R(Pbf)]$_{16}$-Boc) was obtained as red powder. The deprotection cocktail (TFA/TIPS/H_2O 95:2.5:2.5) (1 mL) was added to BTQ-[R(Pbf)]$_{12}$-Boc（186 mg, 0.024 mmol） in a centrifuge tube. After 2 h at room temperature, an orange solid was precipitated by addition of the cool diethyl ether. The precipitate was centrifuged (3500 rpm, 5 min), then diethyl ether was removed by decantation. This operation was repeated twice. The diethyl ether was removed by decantation, and then the orange solid was dried in a vacuum desiccator. The crude product was obtained as orange powder (73 mg, 87%). A part of the crude product was purified by purified by using reversed phase flash chromatography (Biotage) and the following condition: column, SNAP Ultra C18 (Biotage); eluent, 30-100% acetonitrile aq. containing 0.1% TFA. The main peak fraction was collected and purified water was added. The aqueous solution was lyophilized to afford the product (BTQ-R$_{12}$) as orange powder.

ESI-MS (positive): calcd. for $C_{146}H_{228}IrN_{70}O_{16}S_2$ ($[M+4H]^{4+}$): 869.7, found: 869.6.

MALDI-TOF MS: $C_{146}H_{228}IrN_{70}O_{16}S_2^+$: 3474.8, found: 3473.7

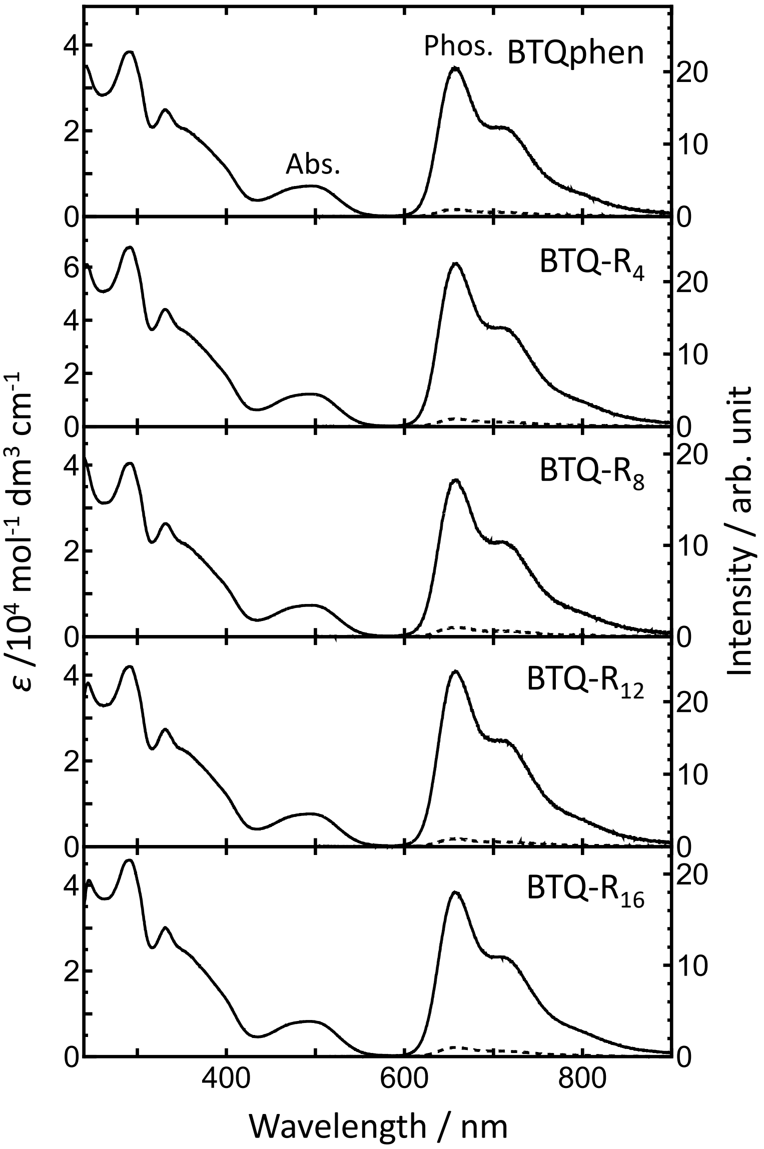

Figure S1. Absorption and phosphorescence spectra of BTQphen and BTQ-R_n (n = 4, 8, 12, 16) in MeCN containing 1% DMSO. Absorption spectra and phosphorescence spectra were measured at room temperatre and 37°C, respectively. The phosphorescence spectra were taken in both deaerated (solid line) and aerated (dashed line) solutions.

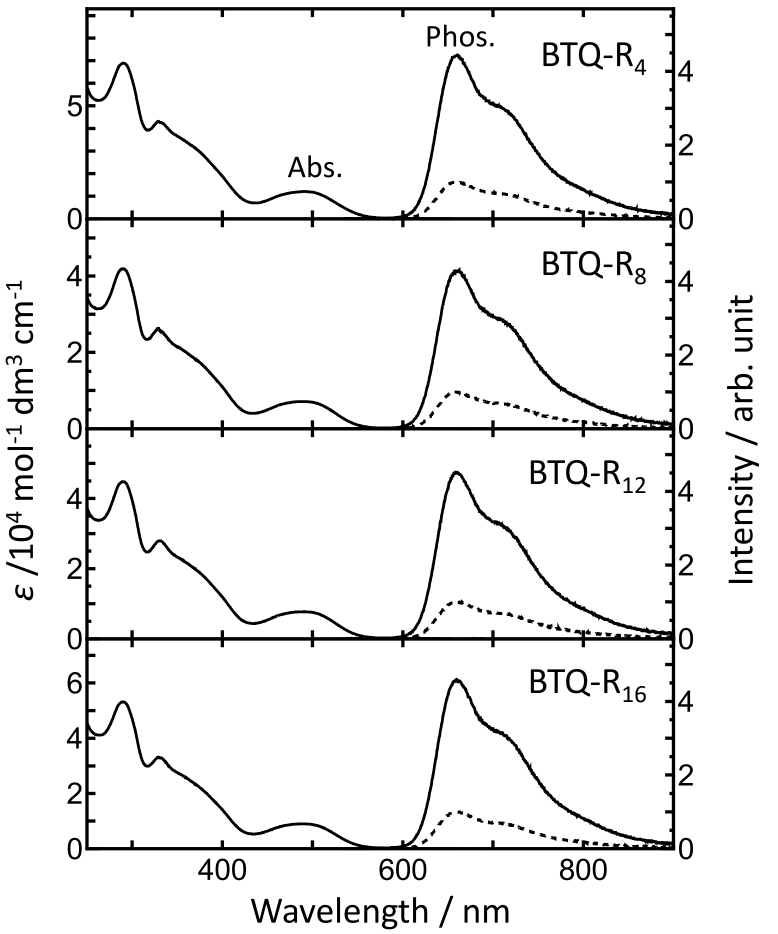

Figure S2. Absorption and phosphorescence spectra of BTQ-R_n (n = 4, 8, 12, 16) in Tris-HCl buffer (pH 7.0) containing 1%DMSO. Absorption spectra and phosphorescence spectra were measured at room temperature and 37°C, respectively. The phosphorescence spectra were taken in both deaerated (solid line) and aerated (dashed line) solutions.

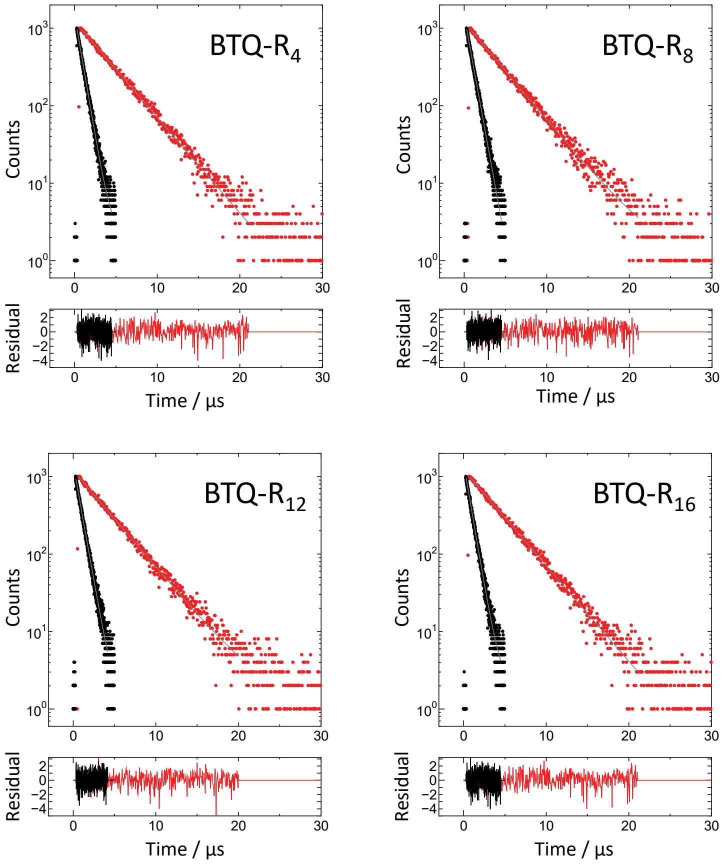

Figure S3. Phosphorescence decay curves of BTQ-R_n (n = 4, 8, 12, 16) in Tris-HCl buffer (pH 7.0) containing 1%DMSO at 37°C. Excited at 488 nm and monitored at 660 nm. Black: aerated, red: deaerated.

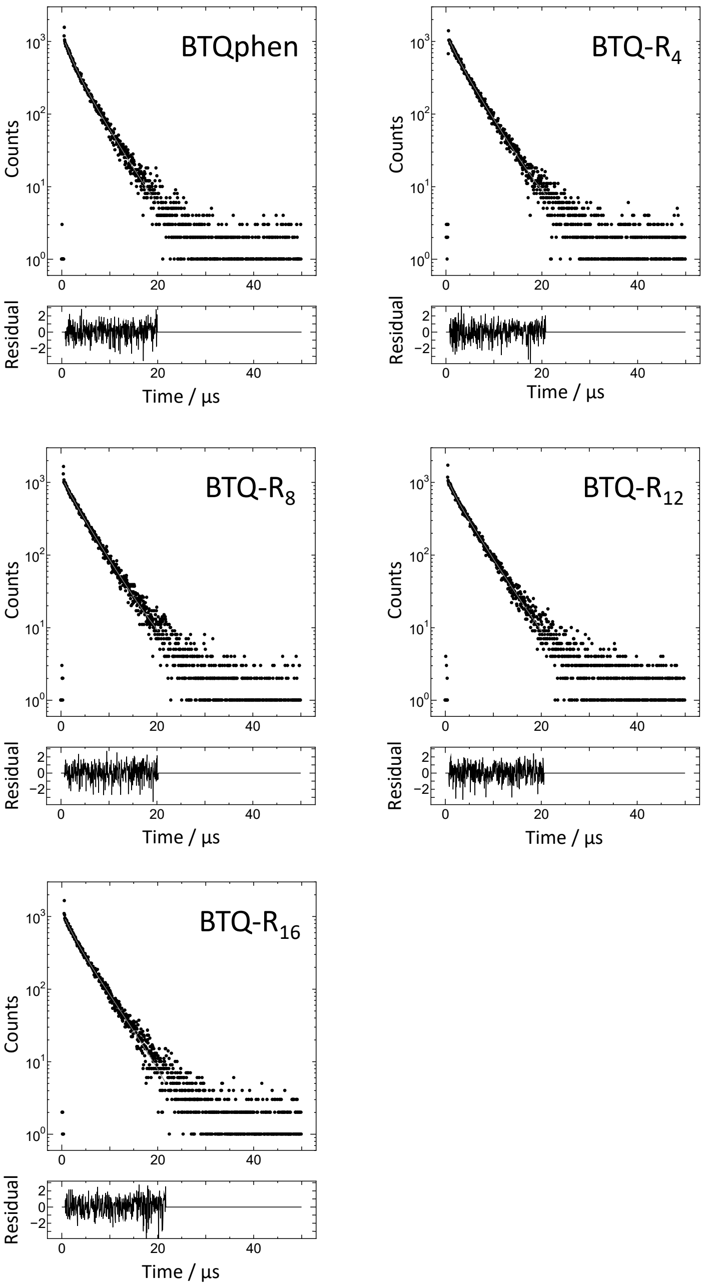

Figure S4. Phosphorescence decay curves of BTQphen and BTQ-R_n (n = 4, 8, 12, 16) in FBS containing 1% DMSO at 37°C. Excited at 488 nm and monitored at 660 nm.

Table S1. Phosphorescence lifetimes of BTQphen and BTQ-R_n (n = 4, 8, 12, 16) in FBS containing 1% DMSO at 37°C. Excited at 488 nm and monitored at 660 nm.

Compound	τ_1/μs	τ_2/μs
BTQphen	1.65 (41%)	4.05 (59%)
BTQ-R_4	1.45 (23%)	4.03 (77%)
BTQ-R_8	1.58 (25%)	4.19 (75%)
BTQ-R_{12}	1.56 (27%)	4.31 (73%)
BTQ-R_{16}	1.57 (24%)	4.23 (76%)

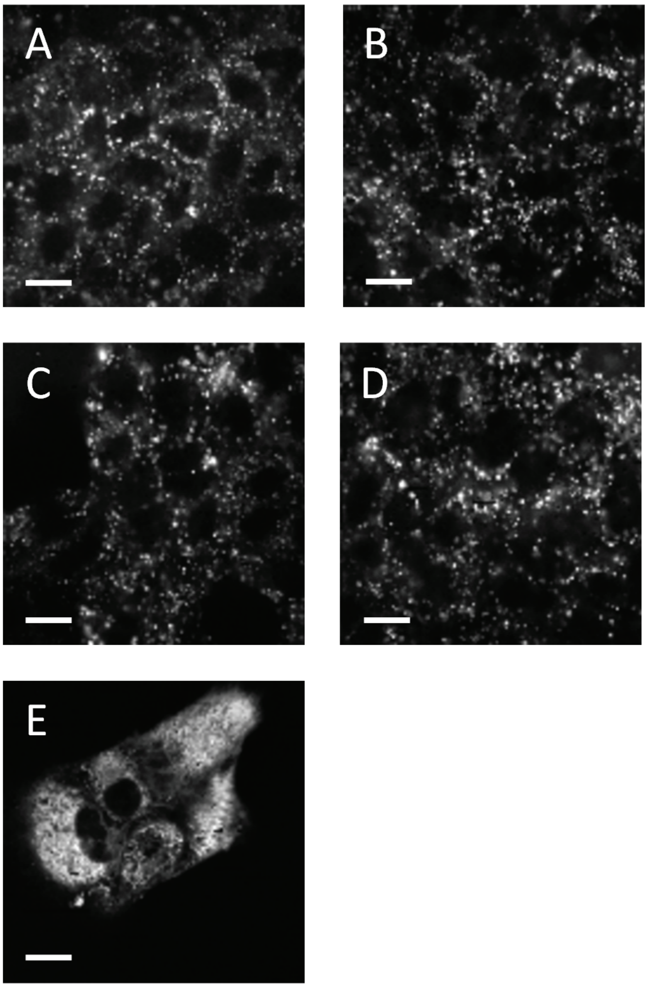

Figure S5. Phosphorescence intensity image of AML12 cells incubated with BTQ-R_4 (A), BTQ-R_8 (B), BTQ-R_{12} (C) BTQ-R_{16} (D), and BTQphen (E) 2 μM for 2 h at 37°C. λ_{exc}: 488 nm, λ_{em}: >590 nm. Scale bar: 20 μm.

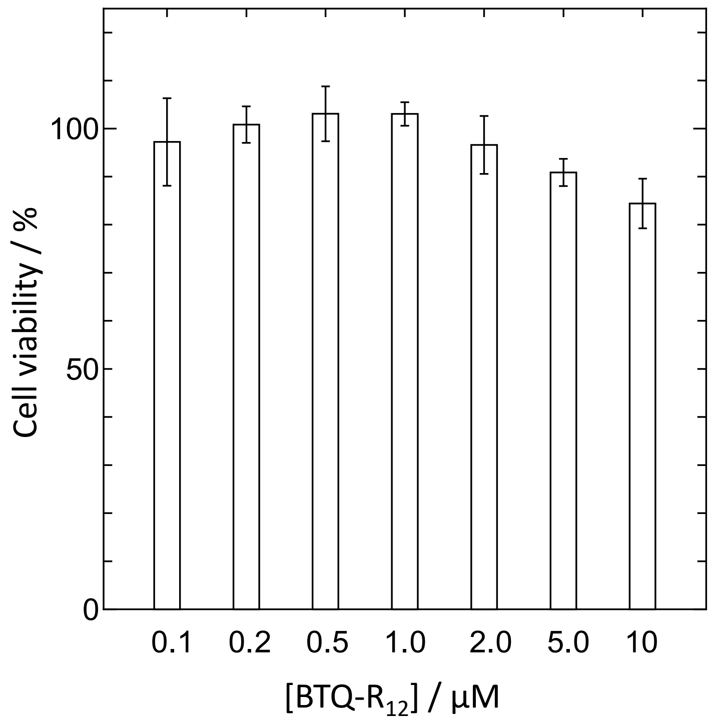

Figure S6. Cell viability of AML12 cells incubated with different concentrations of BTQ-R_{12} for 24 h. Error bars stand for SD (n = 6).

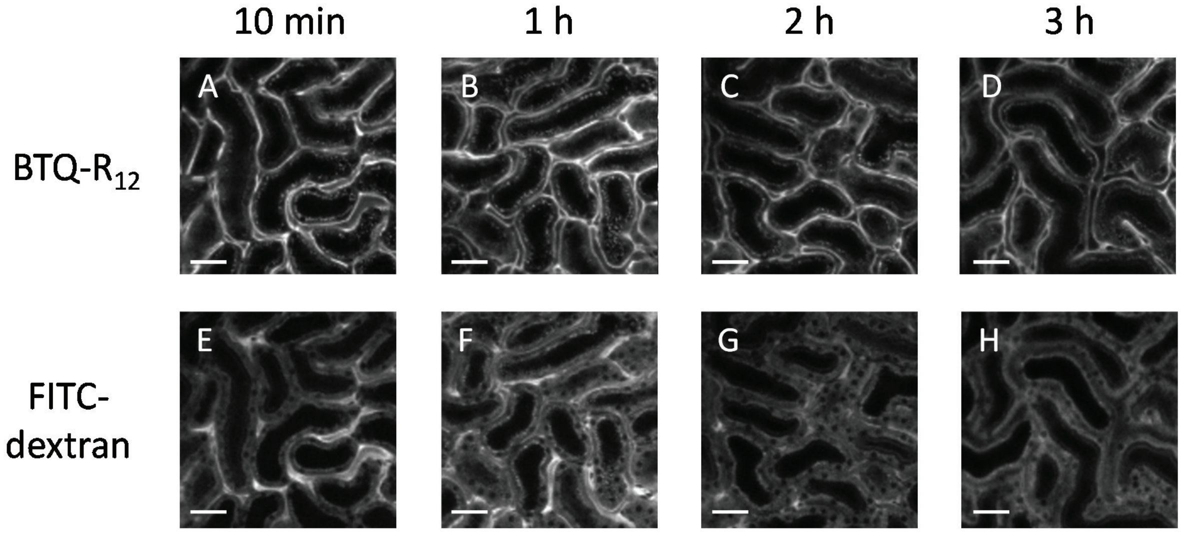

Figure S7. Emission intensity images of kidney observed at 10 min, 1, 2, and 3 h after intravenous injection of BTQ-R_{12} (100 nmol) (A–D) and FITC-dextran (70 kDa) (1 mg/ml, 100μl) (E–H). λ_{exc}: 488 nm, λ_{em}: >620 nm (BTQ-R_{12}), 510-560 nm (FITC-dextran). Scale bar 50 μm.

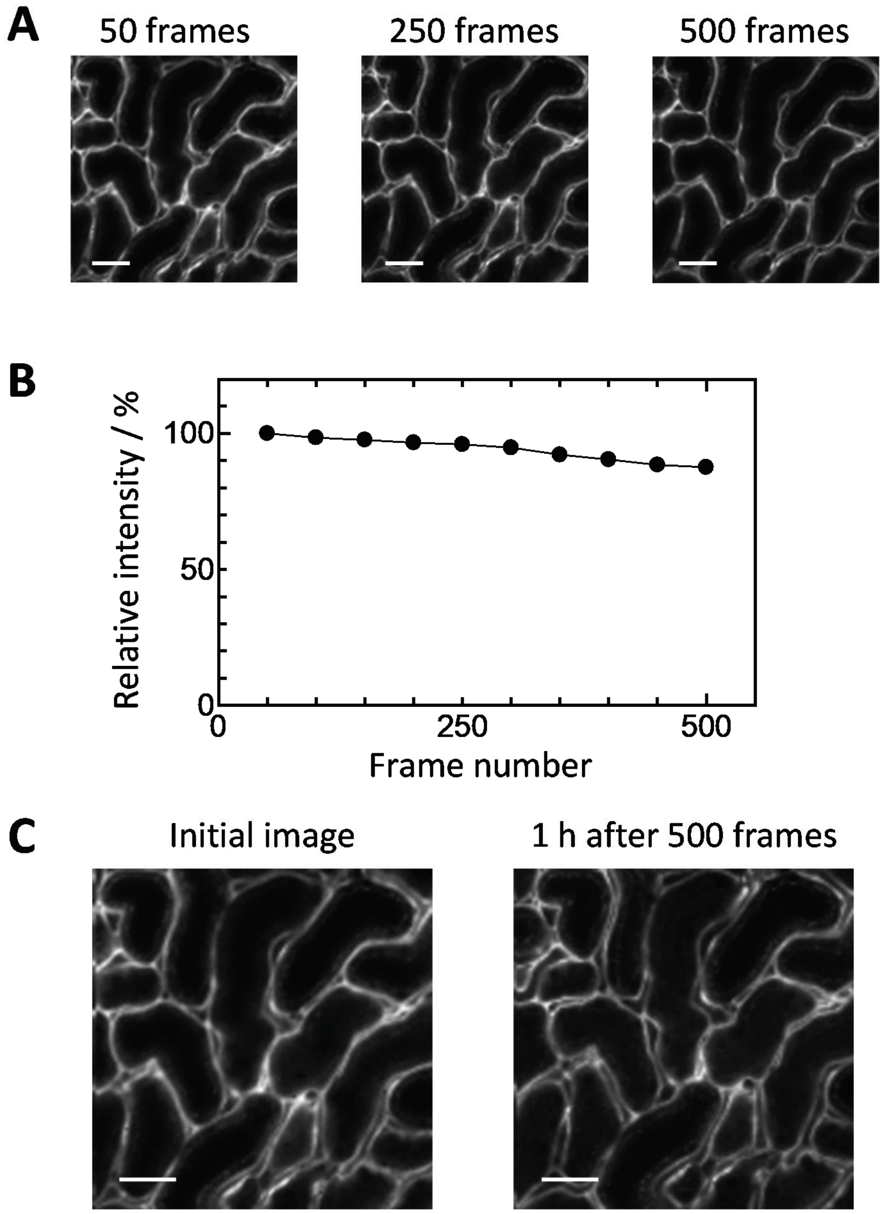

Figure S8. (A) Emission intensity images of renal tissues stained with BTQ-R_{12} (100 nmol). The images were acquired by accumulation over 50, 250 and 500 frames of the scan. (B) Changes in the average intensity of emission images of renal tissue stained with BTQ-R_{12}. (C) Emission intensity images taken by first 50 frames of the scan and after 500 frames of the scan followed by standing for 1 h. λ_{exc}: 488 nm, λ_{em}: >590 nm. Scale bar: 50 μm.

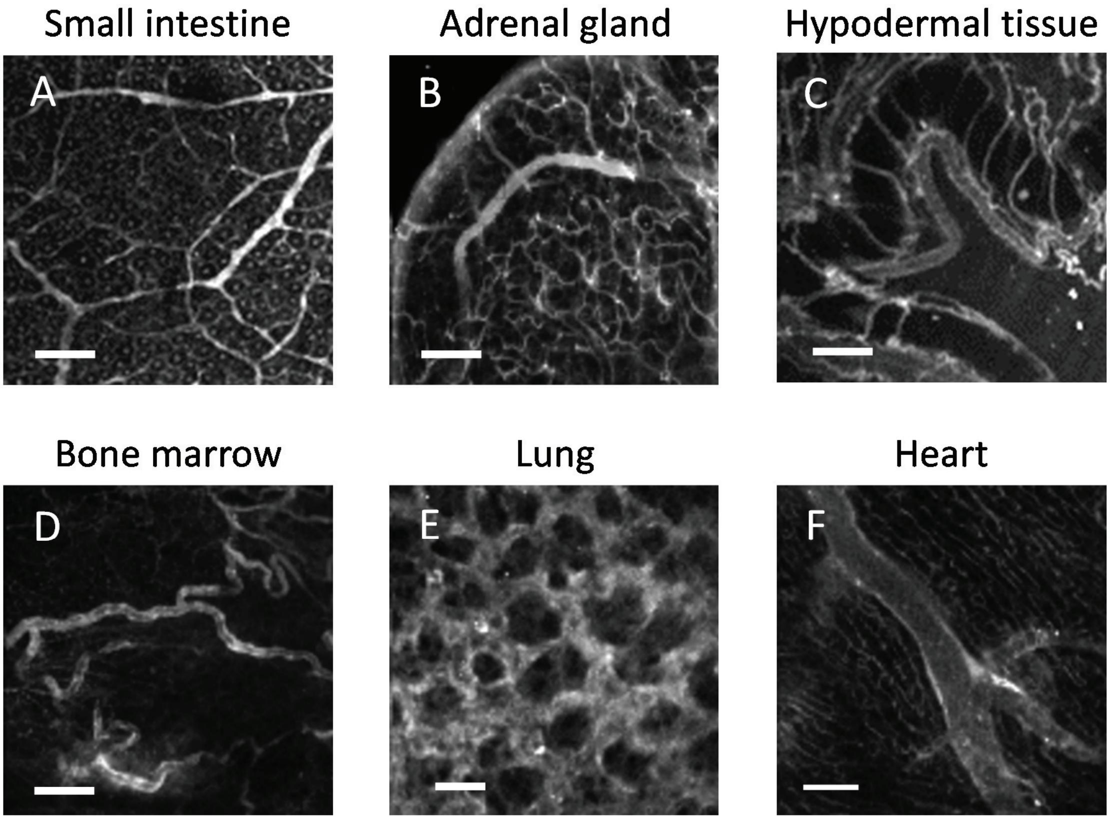

Figure S9. Emission intensity images of small intestine (A), adrenal gland (B), hypodermal tissue (C), bone marrow (D), lung (E), and heart (F). λ_{exc}: 488 nm, λ_{em}: >590 nm. Scale bar: 200 μm in (A), 200 μm in (B), 200 μm in (C), 100 μm in (D), 50 μm in (E), and 100 μm in (F).
